# The Algal Symbiont Modifies the Transcriptome of the Scleractinian Coral *Euphyllia paradivisa* during Heat Stress

**DOI:** 10.3390/microorganisms7080256

**Published:** 2019-08-12

**Authors:** Dalit Meron, Keren Maor-Landaw, Eviatar Weizman, Hiba Waldman Ben-Asher, Gal Eyal, Ehud Banin, Yossi Loya, Oren Levy

**Affiliations:** 1 The Mina and Everard Goodman Faculty of Life Sciences, Bar-Ilan University, Ramat Gan 5290002, Israel; 2 Morris Kahn Marine Research Station, University of Haifa, Haifa 3498838, Israel; 3 School of BioSciences, University of Melbourne, Melbourne, VIC 3010, Australia; 4 ARC Centre of Excellence for Coral Reef Studies, School of Biological Sciences, The University of Queensland, St. Lucia, QLD 4072, Australia; 5 The Institute for Nanotechnology and Advanced Materials, Bar-Ilan University, Ramat Gan 5290002, Israel; 6 Department of Zoology, Tel-Aviv University, Tel Aviv 6997801, Israel

**Keywords:** holobiont, heat-stress, algal symbiont, coral, *Euphyllia paradivisa*, apo-symbiont

## Abstract

The profound mutualistic symbiosis between corals and their endosymbiotic counterparts, Symbiodiniaceae algae, has been threatened by the increase in seawater temperatures, leading to breakdown of the symbiotic relationship—coral bleaching. To characterize the heat-stress response of the holobiont, we generated vital apo-symbiotic *Euphyllia paradivisa* corals that lacked the endosymbiotic algae. Using RNA sequencing, we analyzed the gene expression of these apo-symbionts vs. symbiotic ones, to test the effect of the algal presence on the tolerance of the coral. We utilized literature-derived lists of “symbiosis differentially expressed genes” and “coral heat-stress genes” in order to compare between the treatments. The symbiotic and apo-symbiotic samples were segregated into two separate groups with several different enriched gene ontologies. Our findings suggest that the presence of endosymbionts has a greater negative impact on the host than the environmental temperature conditions experienced by the holobiont. The peak of the stress reaction was identified as 28 °C, with the highest number of differentially expressed genes. We suggest that the algal symbionts increase coral holobiont susceptibility to elevated temperatures. Currently, we can only speculate whether coral species, such as *E. paradivisa*, with the plasticity to also flourish as apo-symbionts, may have a greater chance to withstand the upcoming global climate change challenge.

## 1. Background

Coral reefs are magnificent, massive structures covering ~620,000 square km of the earth [1]. They are the largest structures made by living creatures and contain an enormous diversity of organisms [2]. Reefs support more species per unit area than any other marine ecosystem, making them an important reservoir for biological diversity and complexity [3]. Although coral reefs are classically viewed as ecosystems that exhibit a high degree of stability, ongoing worldwide climate change is threatening to challenge this durability. In the last few decades, we have witnessed dramatic changes in environmental conditions, including increases in temperature, sea level, and ocean acidification, which influence the marine ecosystem as a whole, and corals in particular [4].

Coral–dinoflagellate symbiosis evolved independently several times during the Triassic period (200–250 mya) [5], and today, it is widely accepted that scleractinian coral’s rapid evolutionary success is directly related to the acquisition of endosymbiotic algae [6]. The symbiotic algae provide energy by transferring up to 95% of their photosynthetic production to the host, and in return, derive benefits from the host in the form of nutrients and shelter [7].

Consequently, coral bleaching (loss of the mutualistic intracellular algal cells, and/or loss of photosynthetic pigments) usually leads to changes in coral physiology, and may eventually lead to coral death [8].

Studies investigating the effects of rising sea surface temperatures (SST) (approximately 0.4 °C since the 1950s [9]) have revealed a rise in frequency and intensity of bleaching events, as well as an increase in coral diseases, disorders, and death [10]. Although a number of studies have discussed the global implications and causes of coral bleaching (increased seawater temperature, salinity, ultraviolet radiation, pollution, etc. [11]), there is still a lack of information about how bleaching influences the host’s molecular profile (i.e., gene expression, proteome, biological processes, etc.) and microbial communities.

Gene expression studies have characterized the cellular processes participating in the heat stress response in corals (reviewed by Maor-Landaw & Levy, 2016 [12]). Thermal stress gives rise to oxidative stress as a result of reactive oxygen species (ROS) accumulation. ROS are known to damage membranes, proteins, and DNA in the cell, and their presence is correlated with an up-regulation of antioxidants, defense agents, and heat shock proteins [13,14]. Oxidative stress, in turn, leads to increased calcium concentration and essentially disrupts the calcium homeostasis in the cell, giving rise to a variety of cellular changes including cytoskeleton rearrangement, alterations in the extracellular matrix, cell adhesion disruption, and cell death [15]. ROS and reactive nitrogen species (RNS) can also lead to metabolic alterations in the cell and disruption of oxidative phosphorylation and ATP production in the mitochondria. Under challenge, genes related to energy metabolism are often up-regulated [16,17,18] to satisfy the energetic demands within the cell, but during heat stress, misfolded proteins tend to accumulate in the endoplasmatic reticulum (ER) and trigger the unfolded protein response (UPR), leading to cell cycle arrest and apoptosis [19,20]. Terminally misfolded proteins are marked with ubiquitin for degradation in the proteasome [21,22].

This study focused on the symbiotic mesophotic coral *Euphyllia paradivisa*, which was recently recorded for the first time in the northern Gulf of Eilat (Aqaba), in the Red Sea [23]. *E. paradivisa* is one of 22 scleractinian coral species that are listed as “threatened” by NOAA (National Oceanic and Atmospheric Administration) under the Endangered Species Act (ESA). *E. paradivisa* has shown a continued trend of worldwide loss, decreasing to 38% of previous coverage during the last three decades. Even so, this coral thrives in the mesophotic reefs (36–72 m) and is one of the most abundant species in this depth range, exhibiting up to 30% living coverage of the hard substratum. We chose *E. paradivisa* as a model case study since it exhibits high physiological plasticity, with tolerance to high irradiance and bleaching conditions (the longest ever reported in the literature to survive without algal symbionts in the dark [23]), high competitive abilities, and its photosymbionts exhibited successful adaptation (when corals transferred to shallow water, algal symbiont type was maintained but underwent photoacclimation adjustments) [23].

The aim of this study was to understand whether and how the presence of the algal symbiont (Symbiodiniaceae) influences the holobiont fitness and tolerance to elevated temperatures. The novelty of this project lies in the use of *E. paradivisa* as a model tool to compare the symbiotic and apo-symbiotic coral forms. Previous studies have utilized apo-symbiotic cnidarians, which can be inoculated with algal symbionts, as an ideal mechanism to dissect the specificity of host–symbiont recognition, symbiosis establishment, and maintenance (e.g., Oakley et al. 2016; Lehnert et al. 2014 [24,25]). However, the differential gene expression responses following heat stress have not yet been studied in apo-symbiotic vs. symbiotic corals. To achieve our objectives, we used deep sequencing techniques aligned with various bioinformatics tools to identify specific and common responses of symbiotic and apo-symbiotic *E. paradivisa* to elevated heat stress. This new information may clarify the issue of the contribution of the algal symbiont to host survival when subjected to local or global environmental changes in the marine milieu.

## 2. Materials and Methods

### 2.1. Coral Sampling and Experimental Design

**Figure 1:**
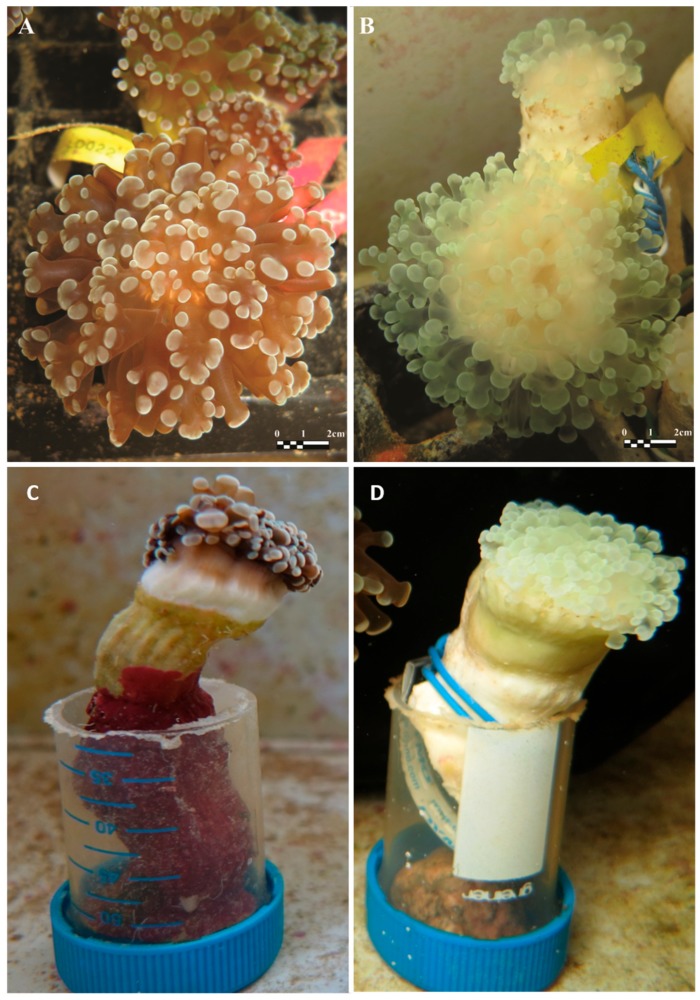
*Euphyllia paradivisa* sub-colonies showing a healthy symbiotic profile (**A**,**C**) and apo-symbiotic sub-colonies (**B**,**D**) after five months in the dark.

Five adult colonies of *E. paradivisa* (20–30 cm) were collected at 45–60 m depth from the Gulf of Eilat (Aqaba, Red Sea) (the Israeli Nature and National Parks Protection Authority approved the collection of corals in this study, permit No 2014/40478). The colonies were fragmented into nubbins of approximately 5 cm, which were then maintained for a period of 12 months in running open-circuit seawater aquaria at the Interuniversity Institute (IUI) for Marine Sciences in Eilat. To generate two sets of morphs—(i) symbiotic polyp (including the natural endosymbiotic algae) and (ii) apo-symbiotic polyp, (lacking the endosymbiotic algae, following bleaching, see Figure 1)—during the 12 months, half of the fragments were maintained under ambient light, while the other fragments were kept in full darkness. The two sets of coral nubbins were grown continuously with running seawater (without feeding), and therefore were naturally fed in a heterotrophic manner.

After the 12 month period, 18 fragments (9 symbiotic and 9 apo-symbiotic polyps) were placed in six aquaria, three tanks for each set. The samples were maintained under a light intensity of 15–52 µmol photons m^−2^ s^−1^, which is characteristic of the conditions in their natural habitat (imitating mesophotic conditions). The ambient seawater temperature was 25 °C and was gradually (~1 °C per day) increased from 25 °C to 28 °C and 31 °C (in practice, we started elevating the temperatures in the 31 °C aquariums, and then, after three days, commenced the increase also in the 28 °C aquariums, to allow the fragments to be maintained in their tested temperature for the same amount of time prior to sampling). After seven incubation days, samples for RNA extraction were collected from the two sets of morphs (symbiotic and apo-symbiotic) at the three different temperatures: 25 °C, 28 °C, and 31 °C. The corresponding samples are referred to as: 25S, 28S, and 31S (symbiotic polyps) and 25AS, 28AS, and 31AS (apo-symbiotic polyps).

### 2.2. Physiological Measurements

During the 12 month growth period in the aquaria, algal symbiont photosynthetic efficiency (*Fv*/*Fm*) was measured under the ambient and dark conditions using a diving PAM (pulse amplitude modulator) fluorometer (Walz, Germany). Monthly measurements were taken in the morning (07:00–08:00), until 2 months after the point at which no further efficiency was detected in the dark-treated samples.

At the end of the 12 months, representative fragments from each colony were taken for chlorophyll a measurements and photosymbiont cells count. Tissue was removed with an airbrush at high air pressure, and host and photosymbiont cells were then separated by centrifugation. Photosymbiont cells were washed three times with 0.22 µ filtered sea water. Chlorophyll a was extracted with 90% acetone and absorbance was measured at 630, 664, and 750 nm with an Ultrospec 2100 pro spectrophotometer (Amersham Biosciences, Switzerland). Chlorophyll a concentration was determined according to Jeffrey and Humphrey 1975 [26] and normalized to photosymbiont cells. Coral tissue volume was determined using the water displacement method [27] before and after tissue removal. Algal cells were counted with a hemocytometer under a microscope and normalized to coral tissue volume.

### 2.3. RNA Extraction and NGS Analyses

To extract RNA, the coral fragments were snap frozen in liquid nitrogen and total RNA was extracted using TRIzol reagent (Invitrogen Life Technologies, Carlsbad, CA, USA) according to the methods presented in Levy et al. 2007, 2011 [28,29], together with a minor modification that included an additional chloroform precipitation and an overnight precipitation in 5 M LiCl at −20 °C. Purified RNA samples were analyzed using a NanoDrop 1000 spectrophotometer (Thermo Scientific) to assess RNA quantity, and a 2100 Bioanalyzer (Agilent, Waltham, MA, USA) to assess RNA quality (RIN > 8.5). Triplicate pools of 1.5 μg RNA (*n* = 6) were prepared using paired-end 300 bp reads from a single Illumina MiSeq run. Overall, six libraries of symbiotic and apo-symbiotic polyps, each at three different temperatures, were run on one lane of an Illumina HiSeq2000 machine using the multiplexing strategy of the TruSeq protocol. On average, ~20–30 million paired-end reads were obtained for each sample. Sequencing was performed at the Next Generation Sequencing (NGS) unit of the Technion research and development foundation LTD (http://isu.thecnion.ac.il), Israel. The Fastq files have been deposited in a SRA database under the accession number: SRP129720.

Trim Galore software (www.bioinformatics.babraham.ac.uk/projects/trim_galore/) was used to trim off sequencing adapters, low-quality reads, and for quality control. The reads were submitted to Trinity software (version r20131110) [30] for de novo assembly using the default parameters. *E. paradivisa* library assemblies resulted in 438,485 contigs (transcripts). Each set of reads from each sample was then aligned to the assembled transcriptome using Bowtie, and RSEM [31] was applied in order to obtain sample-specific abundance estimates. The last two steps were done using the “run_RSEM_align_n_estimate.pl” script from the Trinity software package using the default parameters. To minimize false positive isoforms, all transcripts with an FPKM (fragments per kilobase of exon per million fragments mapped) value < 1 or isoform percentage values (IsoPct%) < 1 were filtered out, and were not included in the downstream analysis. This left 434,603 contigs (N50 = 1353, median contig length = 372). Putative coding regions were extracted from the transcriptome assemblies using TransDecoder software (www.transdecoder.sourceforge.net), providing all the coding sequence (CDS) and proteins from the assembly. The *E. paradivisa* library contained 310,983 open reading frame (ORF)-encoding contigs. Bowtie (v2.1.0) was again used to map each set of reads from each sample against the CDS, and the fold change was calculated based on DESEQ2 (Differential gene expression analysis based on the negative binomial distribution) normalized values [32]. An arbitrary cutoff of at least 1.5-fold (*p*-value < 0.05) was chosen to define a differentially expressed gene (DEG). Annotations were created by blasting the in silico proteome against the *Homo sapiens* database using reciprocal BLASTP (NCBI). After annotation filtration, 5580 annotated contigs remained.

### 2.4. Bioinformatics Analysis

We utilized Partek Genomic suite software (version 6.6, Partek Inc., St. Louis, MO, USA) to plot a three-dimensional space principle component analysis (PCA) and to generate Venn diagrams and hierarchical clustering heat maps for *E. paradivisa* samples (heat maps were generated using Pearson distance as a similarity measure and average linkage). Hierarchical clustering dendrogram with p-values was done using the R module pvclust, with multiscale bootstrap resampling of 1000 iterations to assess statistical significance, represented by a 1–100 score. Clustering was done using the average method with correlation as the distance metric.Functional gene analysis was done by David Bioinformatics Resources 6.8 [33] using the *Homo sapiens* ortholog annotations. The alternative of aligning to a closer species resulted in poor annotations, and their functionality was also not well known, in contrast to the counterparts in the *Homo sapiens* database.

We utilized a list of cnidarian environmental stress genes derived from the relevant literature [12] to search the *Homo sapiens* database annotations and identified DEGs. Further, for every annotation, we determined if the fold changes of the treatments were in correlation to the morph or, alternatively, to the temperature treatment. Additionally, we marked which samples followed the trend reported in the literature. Pearson correlation was calculated using Free Statistics Software [34] to assess the correlation between the fold change values of our treatments with the literature values. In a similar manner, a list of symbiosis differentially-expressed genes was generated based on symbiotic vs. apo-symbiotic experiments in cnidarians [24,25,35,36,37], and was utilized to find relevant DEGs in our database. Furthermore, Fisher’s exact test was performed in an attempt to estimate which samples were enriched with “symbiosis genes.” These data were utilized to create pie charts showing those parameters in a quantitative manner as percentages of total identified genes.

## 3. Results

*Euphyllia paradivisa* polyps were kept in completely dark conditions for a year, generating an apo-symbiotic morph. After the first five months, *E. paradivisa* sub-colonies lost their original color and became totally white (Figure 1), and at the completion of the twelve months in the dark, no algal cells and chlorophyll a were detected (as opposed to the fragments under light conditions, in which 1.34 × 10^8^ ± 6.43 × 10^7^ algal cell and 17.12 ± 8.57 chlorophyll per ml^−3^ (µg/ml^−3^) were measured) (Table S1). The fluorescence yield measured using PAM fluorometry showed that the *Fv/Fm* values of the bleached polyps decreased to undetectable values of less than 10% efficiency, whereas the polyps that were grown under natural light regime gave constantly high values of photosynthetic efficiency (*Fv*/*Fm* of ca. 0.7). Despite the lack of symbiotic algae in the apo-symbiotic polyps, they remained vital for the whole year, with fully extended tentacles and low mortality rates. Additionally, no mortality was detected during the thermal stress experiment [23]).

**Figure 2:**
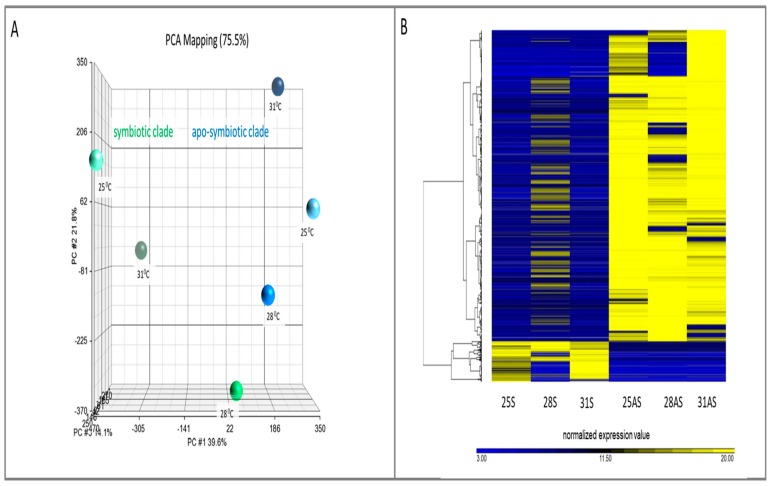
Clustering of symbiotic and apo-symbiotic samples. (**A**) Principle component analysis (PCA) of all the samples, capturing 75.5% of total variability. (**B**) Heat map of hierarchical clustering of all differentially expressed genes. S: symbiotic; AS: apo-symbiotic; 25, 28, and 31: temperature treatment. Scale shows normalized expression values generated using Euclidean distance as a similarity measure and average linkage.

*E. paradivisa* sequenced reads were assembled into a transcriptome of 434,603 open-reading-frame contigs. A hierarchical clustering dendrogram with p-values was done on the expression values of these genes confirming the segregation of the symbiotic and apo-symbiotic into two groups (Figure S1). Figure 2A displays the variance of the samples, as it captured 75.5% of the total variability for different temperatures and morphs, and the hierarchical clustering heat map. Figure 2B presents this differential gene expression pattern on a color scale. Figure 2 shows that the symbiotic and apo‑symbiotic samples are segregated into two groups. Moreover, the PCA (principal component analysis; Figure 2A) detected that the 31 °C temperature and the 25 °C control samples were grouped more closely within the symbiotic cluster, and were somewhat separated from the 28 °C samples.

**Figure 3:**
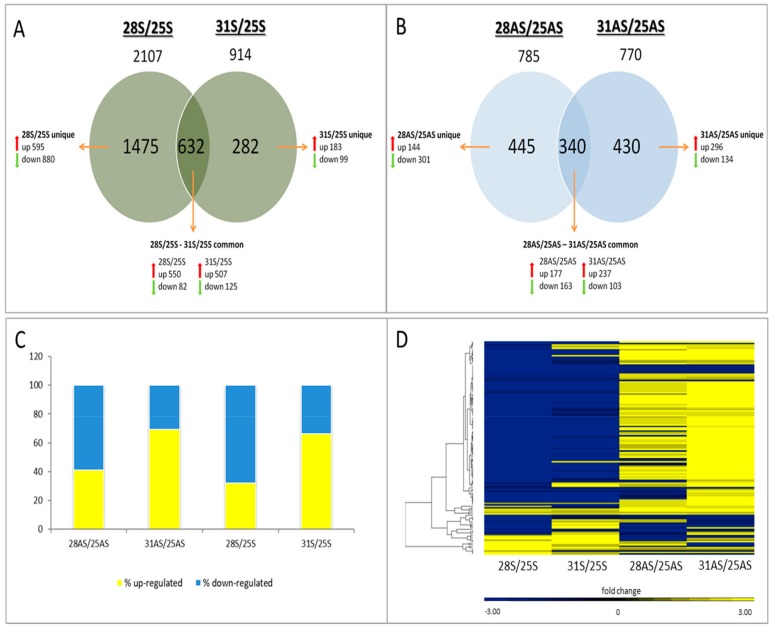
Numbers and expression patterns of differentially expressed genes. (**A**,**B**) Venn diagrams of symbiotic and apo-symbiotic samples, respectively. The numbers of up- and down-regulated genes are indicated within each field with regard to unique and common groups. (**C**) Percentage of up- and down-regulated genes in the treatments. (**D**) Hierarchical clustering heat map of fold change of 139 genes that were common amongst the four treatments. S: symbiotic; AS: apo-symbiotic; 25, 28, and 31: temperature treatment.

In addition to the DESEQ analysis, normalized values from each temperature treatment were compared to those of the relevant morph control (28S and 31S to 25S; 28AS and 31AS to 25AS). We constructed Venn diagrams (Figure 3A,B) of all differentially expressed genes (both up- and down-regulated), highlighting common and unique genes among the temperature treatments and symbiotic and apo-symbiotic *E. paradivisa*. In general, the symbiotic samples yielded higher numbers of DEGs than the apo-symbiotic samples, and the 28S/25S comparison had the highest number (2107 genes). The percentage of up- vs. down-regulated genes showed a temperature-dependent trend (Figure 3C): at 28 °C, most of the DEGs were down-regulated (both in the symbiotic and apo-symbiotic morphs), whereas at 31 °C, most of the DEGs were up-regulated (both in the symbiotic and apo-symbiotic states).

A total of 139 differentially expressed genes were identified in all four treatment samples and morph types, and these were termed “common genes” (Figure S2). Moreover, a hierarchical clustering heat map of these common genes demonstrates that the trend of their gene expression pattern was, in most cases, opposite between symbiotic and apo-symbiotic *E. paradivisa*, such that in the apo-symbiont most were up-regulated, while in the symbiont state they were down-regulated (Figure 3D).

**Figure 4:**
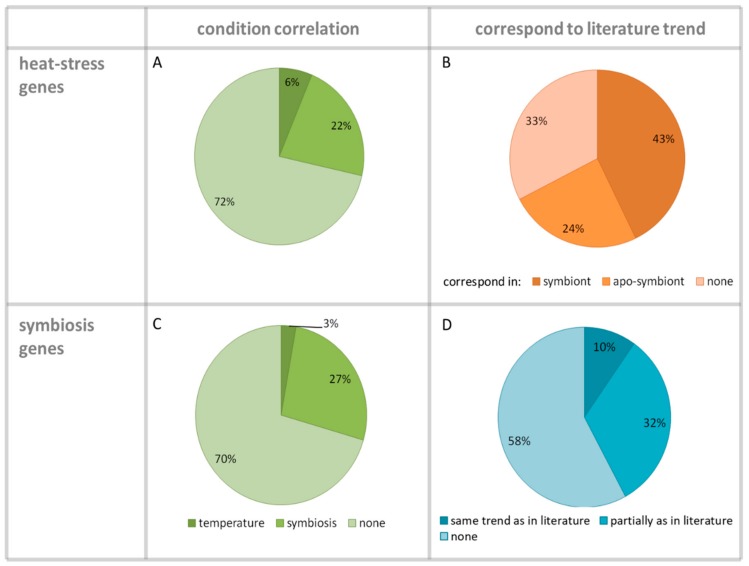
Condition correlation and correspondence with literature trend of “heat-stress genes” and “symbiosis genes.” Percentages are shown out of the total identified 126 and 71 genes of heat stress and symbiosis, respectively. (**A**,**C**) Percentage of genes categorized by the condition (temperature/symbiosis) correlation regarding “heat-stress genes” and “symbiosis genes,” respectively. A gene with a fold change trend similar in 28S–28AS and in 31S–31AS was defined as: “temperature-correlated,” while a gene with a fold change trend similar in 28S–31S and in 28AS–31AS, was termed: “symbiosis-correlated.” (**B**) Percentage of “heat-stress genes” that corresponded to the trend in the literature for symbiont and apo-symbiont treatments. (**D**) Percentage of “symbiosis genes” that followed the trend as described in the literature. When just one symbiotic or apo-symbiotic sample followed the trend, it was defined as ‘partially as in literature.’

This study considered two independent variables/parameters: temperature elevation (28 °C/31 °C) and the presence or absence of the photosymbionts. Therefore, we conducted a comprehensive literature survey of genes known to be differentially expressed under these conditions for comparison with our current data. As a result of this survey, we prepared a list of coral heat-stress genes [12] to be used to search the annotations. We identified 126 heat-stress genes, the characteristics of which (fold changes and trend) are summarized in Table S2. Analysis of this table revealed that 22% of the genes had a differential expression pattern that was correlated with the morph type (Figure 4A), meaning that the symbiont samples 28S/25S and 31S/25S behaved similarly, as did the apo-symbiont pairs 28AS/25AS and 31AS/25AS. Merely 6% of the heat-stress differentially-expressed genes correlated with the temperature condition, meaning they exhibited a similar trend in the pairs 28S/25S and 28AS/25AS and a different one in the pairs 31S/25S and 31AS/25AS. We compared the documented trends in the literature to our results and estimated that 43% of the genes could be recognized in the symbiotic samples, while this value was reduced to 24% when considering the apo-symbiotic samples (Figure 4B). Moreover, the Pearson correlation calculated showed a correlation between the 28S treatment and the literature values (two-sided *p* < 0.05) in the same direction. It is noteworthy that the list of heat-stress genes was derived from experiments conducted on symbiotic cnidarians; thus, there is an uncertainty regarding the gene expression responses of apo-symbiotic cnidarians to heat stress. This might explain our results showing a greater similarity between the literature and the gene expression data of the symbiotic samples.

A list of “symbiosis differentially-expressed genes,” which was based on relevant studies, was used to search our annotation data and identified 71 differentially expressed genes (Table S3). Fisher’s exact test showed that both symbiotic and apo-symbiotic samples were enriched with “symbiosis genes,” but that the *p*-value was much more significant for the symbiotic samples (2-tail *p*-value = 3.2 × 10^−17^, compared to 3.1 × 10^−7^ for the apo-symbiotic samples). Our results demonstrate that, again, the expression of the “symbiosis genes” was determined more by morph state than by temperature. The expression of 27% of the genes was correlated to the presence of the photosymbiont, with only 3% related to the temperature (Figure 4C). It should be noted, however, that the trend of most of these genes (up- or down-regulation) was not necessarily in accordance with the literature reports (Figure 4D). One reason for this inconsistency could be that most of the “symbiosis genes” have been derived from experiments conducted on sea anemones, as model organisms to study corals, which possibly exhibit some different trends with or without symbionts due to different life histories (i.e., calcification). Moreover, both datasets, heat-stress genes and symbiosis genes, were mostly based on shallow-water cnidarians, and the gene expression response of a mesophotic coral might be different.

**Figure 5:**
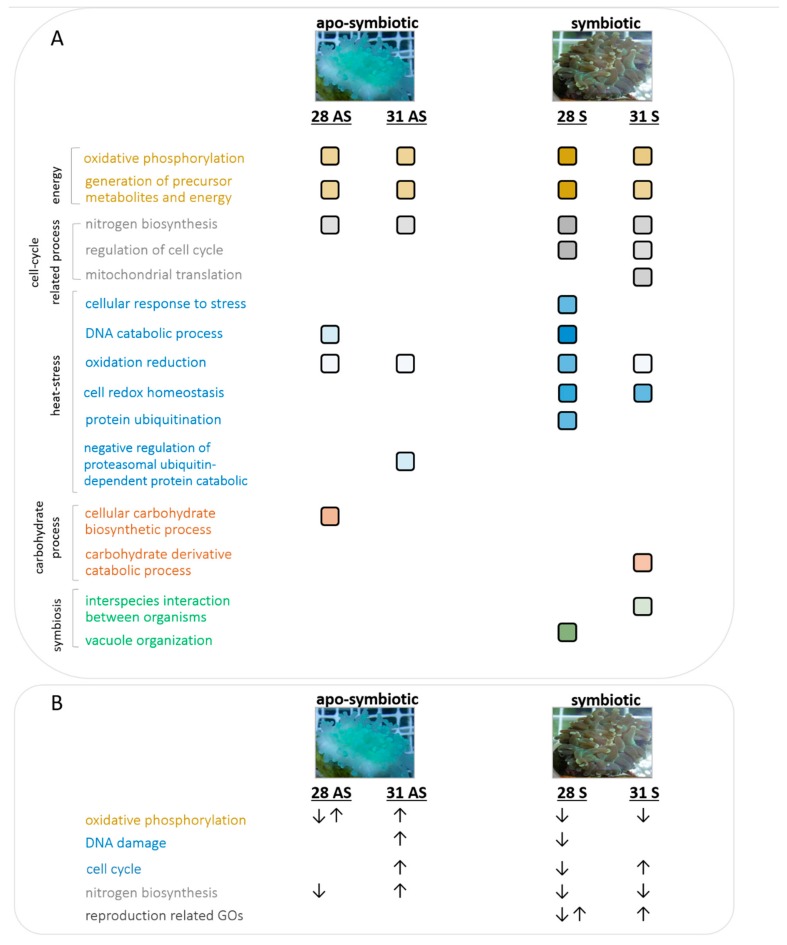
A model summarizing enriched cellular processes and their trends. (**A**) Enriched gene ontologies in symbiotic and apo-symbiotic *E. paradivisa* throughout the experiment. The color intensity gradient of the squares represents the enrichment of the GO in the treated samples. (**B**) Up- and down-regulated trends of enriched categories in 31 °C-treated apo-symbiotic and 28 °C-treated symbiotic *E. paradivisa.*

A model summarizing the selected enriched gene ontologies (GO) in symbiotic and apo-symbiotic *E. paradivisa* in the different treatments in the experiment is presented in Figure 5. Panel B in Figure 5 presents the different directions (up-vs. down-regulation) of certain GOs. Processes that are related to increased cellular energy and metabolites appeared to be more dominant at 28 °C in the symbiotic morphs. This treatment also displayed the highest number of GOs known to be common in heat-stress scenarios, including protein ubiquitination, DNA catabolism, and oxidation–reduction. Cellular carbohydrate biosynthesis was enriched in the mild temperature (28 °C) treatment in the apo-symbiotic coral, while catabolism was significant at 31 °C in the symbiotic state. Not unexpectedly, processes related to symbiosis, such as interspecies interactions (the symbiosome is a vacuole derived from early endosome), were enriched only in the symbiotic and not in the apo-symbiotic morph. Moreover, GOs related to meiosis and reproduction were found to be unique to the symbiotic coral, and occurred only in the mild temperature treatment.

## 4. Discussion

In this study, we analyzed the gene expression response following thermal stress exploring the effect of an algal symbiont presence on the transcriptome of the coral. The novelty of our case study approach lay in utilizing symbiotic and apo-symbiotic *E. paradivisa* corals as a model of a relatively tolerant coral [23]. Until now, studies of symbiotic and apo-symbiotic cnidarians have been designed to reveal symbiosis specificity and maintenance, and have not been utilized for a better and necessary understanding of corals under the threat of global climate change. One of our key findings was that the presence of algal symbionts greatly influences the coral host’s gene expression pattern, indeed, much more than the environmental temperature conditions that the holobiont is subjected to. This was demonstrated unambiguously in the PCA and hierarchical clustering (Figure 2), which indicated two distinct clusters: symbiotic *E. paradivisa* and apo-symbiotic *E. paradivisa* samples. Moreover, the analysis of specific “symbiosis genes” and “heat-stress genes” supported this general trend; the differential expression of at least three times more genes could be correlated to the morph state than were related to the temperature treatment. Thus, the nature of the gene expression response to thermal stress would likely be determined firstly by the coral symbiotic state and presence, and not by the stringency of the heat.

The identified 139 differentially expressed genes that were apparent in all treatments (common genes), constituted a general response to the heat, and were not restricted to specific temperature or morph. Interestingly, they behaved differently in symbiotic and apo-symbiotic corals (mostly down- and up-regulated in symbiotic and apo-symbiotic, respectively). This result further strengthened our finding of symbiotic and apo-symbiotic differential gene expression response to thermal stress. In addition to the common genes, there were differences in gene expression between different temperatures and morphs.

In the symbiotic corals, the milder 28 °C temperature triggered the strongest gene expression response (samples 28S). The number of differentially expressed genes in the 28S samples was almost three times higher than in the apo-symbiotic preparations (Figure 3A,B). However, the response was in the form of acute shut down of genes, which may be the result of a cellular shock in the symbiotic *E. paradivisa*. Increasing the temperature to 31 °C appeared to moderate the gene expression response to the heat, in terms of number of genes and respective GOs, which was mostly diverted into up-regulation of genes. This result might indicate a severe cellular state, where the mechanisms to cope with the stress by activating stress genes are overwhelmed and irresponsive in a way. The observation of a higher number of DEGs at 28 °C than at 31 °C was also documented by RNA microarray analysis for the Red Sea coral *Stylophora pistillata* [16]. Another example where the heat-stress response (DEGs numbers) was not directly correlated with the time/temperature of coral exposure was described by Seneca and Palumbi (2015). These researchers reported that the transcriptome response of *Acropora hyacinthus* colonies was more significant after 5 hours of exposure to heated conditions than after 15 h [38]. The heat map of hierarchical clustering (Figure 2B) also supported this DEG behavior, as the 31S and 25S samples were more comparable to each other than either one was to the 28S sample. This pattern identified the peak of the stress reaction in the symbiotic coral as 28 °C, with remission at 31 °C. The peak temperature seems to be a critical threshold for this mesophotic coral, as in its natural habitat in the Red Sea, maximum summer temperatures do not exceed 28 °C [39].

Considering this behavior, it is not surprising that the 28S samples represented those most enriched for GOs (Figure 5A). Not only did the 28S samples contain up to six times more categories of GOs than any of the other treatments, but there were also more genes in each enriched category (thus, more significant). More specifically, there were two heat-stress-related GOs that were apparent only in the 28S samples; “cellular response to stress” and “protein ubiquitination.” Down-regulation of cell-cycle-associated genes in the 28S samples could indicate that the cells were in the process of cell cycle arrest or even cell degradation. Though all the treatments probably caused cellular oxidative stress (GO: cell redox homeostasis, oxidation-reduction), the symbiotic coral apparently suffered the strongest stress at 28 °C, as evidenced by the finding that the cellular response to stress was escalated to protein degradation (GO: protein ubiquitination) and to DNA damage (GO: DNA damage).

Our results imply that not only did the presence of the photosymbiont influence the coral’s thermal resilience, but that it had a negative effect, as can be seen in both the “heat-stress genes” analysis (Figure 4B) and in the GO model (Figure 5). Analysis of GOs demonstrated down-regulation of oxidative phosphorylation in both the 28S and 31S samples (Figure 5B). This is a common outcome of environmental stress, which disrupts ATP production in the mitochondria [12]. We postulate that the increased susceptibility of symbiotic *E. paradivisa* to oxidative stress may be largely attributed to the ROS-producing algal symbionts within their gastrodermal tissues. Following thermal stress, the chloroplasts of the photosymbiont may represent the prime source for ROS [13]. In fact, coral bleaching has been suggested as a survival technique, where the host expels the compromised ROS-producing photosymbionts and the symbiosis breaks down [40,41]. We have shown that in the massive *Porites* sp., the expression of stress genes was postponed after bleaching. Thus, it was suggested that expelling the algal symbionts enabled mitigation of the oxidative damage so that the associated stress genes could be activated at a later stage [42]. It is possible to postulate that a coral that can be vitally sustained as an apo-symbiont may be more resilient to thermal stress in that state. Some coral species have the capability as apo-symbionts to switch from acquiring fixed carbon primarily photoautotrophically to relying on alternative sources of fixed carbon: heterotrophic feeding and use of stored energy reserves [43]. Our experiments have shown that *E. paradivisa* has a heterotrophic plasticity required for long term durations without algal symbionts, and thus raise the question of whether the *E. paradivisa*–dinoflagellate relationship is more of a facultative nature, rather than obligatory. However, further research is needed in order to prove or disprove these hypotheses.

Our results are also in agreement with the hypothesis proposed by Caroselli et al. [44,45] that non-symbiotic (asymbiotic) scleractinians may be more tolerant to temperature increase than symbiotic varieties. They measured variations in biometric parameters (polyp size, linear extension, calcification rate, skeletal mass, and skeletal density) and population density of symbiotic and asymbiotic corals along a latitudinal sea surface temperature (SST) gradient on western Italian coasts (Mediterranean Sea). While the asymbiotic coral seemed unaffected by SST and the biometric parameters did not change between the sites, the parameters of the symbiotic coral were inversely correlated to SST. The higher tolerance of the asymbiotic coral was attributed to the absence of photosymbionts, and thus freedom from inhibition of host physiological processes by the heat-stressed algal cells [44,45].

Several GOs were differentially enriched in symbiotic versus apo-symbiotic corals. Cellular carbohydrate biosynthetic processes were found to be enriched in the mild temperature apo-symbiotic samples (Figure 5), which was probably related to metabolic changes in the apo-symbiotic host resulting from the lack of photosynthates regularly transferred from the phototrophic symbiont in symbiotic corals [46]. However, the pathways resulting in the breakdown of carbohydrate derivatives were enriched in the symbiotic morphs (31S), which benefited from the photosynthetic products translocated from the algal endosymbiont. Symbiosis-related GOs appeared only in symbiotic *E. paradivisa* samples, but, for some reason, these were temperature-dependent and were not consistent in the 28S and 31S samples (Figure 5). Enrichment of the vacuole organization GO could be explained by the fact that algal symbiont is maintained distinct from the host cytoplasm within a ‘symbiosome,’ which is a host-derived vacuole [47]. Another set of GOs that were enriched only in the symbiotic samples and absent from the apo-symbiotic were those associated with reproduction and meiosis. These results agree with studies showing that bleaching profoundly affects the reproductive characteristics of corals (i.e., reduced numbers of eggs and testes, egg morphometry, and sperm motility) in both the short- and long-term [48,49,50].

Studies on gene expression responses of corals to environmental stress have largely focused on shallow-water corals [12]. Nevertheless, understanding these processes in mesophotic corals is crucial as well, given the forecasted degradation of shallow-water reef habitats [4]. Mesophotic coral reef ecosystems have been hypothesized to serve as a refuge for shallow and mid-depth species from various environmental disturbances (‘deep reef refuge’ hypothesis) [51,52]. However, to the best of our knowledge, there has been no experiment that has compared the tolerance of shallow-water and mesophotic corals to elevated temperatures under a controlled system. The scenario where mesophotic corals can thrive in the reef without their algal symbionts, possibly calcify their skeletons at a slow rate [53,54], be less susceptible to elevated temperatures, and regain the symbionts when conditions allow it, should be further explored in future studies.

## 5. Conclusions

Overall, our results suggest that the presence of algal symbionts can impair coral holobiont susceptibility to elevated temperatures. At the present stage, we can only speculate whether coral species with the plasticity to also flourish as apo-symbionts, such as *E. paradivisa*, may have a greater chance to withstand the upcoming global climate change challenge. Future studies should consider looking deeply into the effects of global climate change on the holobionts of symbiotic and apo-symbiotic corals.

## Supplementary Materials

The following are available online at https://www.mdpi.com/2076-2607/7/8/256/s1, Figure S1: Hierarchical clustering dendrogram with p-values. Values on the edges of the clustering are p-values (%). Hierarchical clustering dendrogram with p-values was done using the R module pvclust (Suzuki & Shimodaira, 2006). Red values are AU (Approximately Unbiased) p-values, and green values are BP (Bootstrap Probability) values, Figure S2: Venn diagram of S; symbiotic polyps and AS, apo-symbiotic common genes. The diagram includes only the common genes from each treatment (340 common genes of apo-symbiotic and 632 common genes of control groups, see Figure 3). The numbers of up- (red arrows) and down- (green arrows) regulated genes are indicated within each field, as regard to the examined groups 28AS/25S, 31AS/25AS 28S/25S, and 31S/25S. Figure includes data of number of genes in common and unique groups, Table S1: Number of symbiont cells and chlorophyll measurements in representative fragments of each treatment, following one year in light/darkness conditions. Table S2: Summary of identified environmental stress genes in the treatments. Indicated in the table: the HS annotation, fold change of the four treatments (28/31: temperature of exposure in 0C, AS; apo-symbiotic polyps, S: symbiotic polyps, in yellow: treatments that follow the trend in the literature), annotated protein name, the trend in the literature (up/down-regulated), correlation (purple; the treatments’ fold change is in correlation to the morph, green; the treatments fold change is in correlation to the temperature treatment), and which morph corresponds with the trend in the literature, Table S3: Summary of identified symbiosis genes in the treatments. Indicated in the table: reference, annotated protein name in the reference, process in the cell, HS annotation, fold change of the four treatments (28/31: temperature of exposure in 0C, AS: apo-symbiotic polyps, S: symbiotic polyps), annotated protein name, the trend in the reference (up/down-regulated/unique in S: symbiotic/AS: apo-symbiotic cnidaria, yellow: our results are in the same trend as in the paper, orange; our results are partially in the same trend as in the paper), and correlation (purple: the treatments’ fold change is in correlation to the morph, green; the treatments fold change is in correlation to the temperature treatment).
