# Supplementary material for: The Algal Symbiont Modifies the Transcriptome of the Scleractinian Coral Euphyllia paradivisa during Heat Stress
Source: Microorganisms. 2019 Aug 12;7(8):256. doi: 10.3390/microorganisms7080256 (PMC6723837; doi:10.3390/microorganisms7080256)
Supplement: Supplementary file 1 [file microorganisms-07-00256-s001.zip › Meron et al_microorganisms_SI_proofed2.docx]

**Supplementary material for**

The algal symbiont modifies the transcriptome of the scleractinian coral *Euphyllia paradivisa* during heat stress

Meron Dalit ^1,5,†^, Maor-Landaw Keren ^1,6,†^, Weizman Eviatar ^1^, Waldman Ben-Asher Hiba ^1^, Eyal Gal ^1,7^, Banin Ehud ^1,4^, Loya Yossi ^2^ and Levy Oren ^1,^*

^1^ The Mina and Everard Goodman Faculty of Life Sciences, Bar-Ilan University, Ramat Gan 5290002, Israel

^2^ Department of Zoology, Tel-Aviv University, Tel Aviv  6997801, Israel

^3^ The Interuniversity Institute for Marine Sciences of Eilat, Eilat 8810302 Israel

^4^ The Institute for Nanotechnology and Advanced Materials, Bar-Ilan University, Ramat Gan 5290002, Israel

^5^ Morris Kahn Marine Research Station, University of Haifa, Haifa 3498838, Israel

^6^ School of BioSciences, University of Melbourne, Melbourne, VIC 3010 Australia

^7^ ARC Centre of Excellence for Coral Reef Studies, School of Biological Sciences, The University of Queensland, St. Lucia,,Qld 4072 Australia

^†^ These authors contributed equally to this work

***** Correspondence: [oren.levy@biu.ac.il](mailto:oren.levy@biu.ac.il); Tel: +97235318030, Fax: +97237384181

*These authors contributed equally to this work

Meron Dalit; dalitmeron@gmail.com, Maor-Landaw Keren; [keren.maor@live.com](mailto:keren.maor@live.com), Weizman Eviatar; eviatar.weizman@gmail.com, Waldman Ben-Asher Hiba; hba151@gmail.com, Eyal Gal; g.eyal@uq.edu.au, Banin Ehud; ehud.banin@biu.ac.il, Loya Yossi; yosiloya@post.tau.ac.il, Levy Oren; Oren.Levy@biu.ac.il

Corresponding author – Oren Levy; email: [oren.levy@biu.ac.il](mailto:oren.levy@biu.ac.il)

Bar-Ilan University Ramat Gan, 5290002 Israel

tel. +97235318030, fax +97237384181

Running title: symbiont modifies transcriptome of heat stressed coral

Figure S1 - Hierarchical clustering dendrogram with p-values. Values on the edges of the clustering are p-values (%). Hierarchical clustering dendrogram with p-values was done using the R module pvclust (Suzuki & Shimodaira, 2006). Red values are AU (Approximately Unbiased) p-values, and green values are BP (Bootstrap Probability) values.


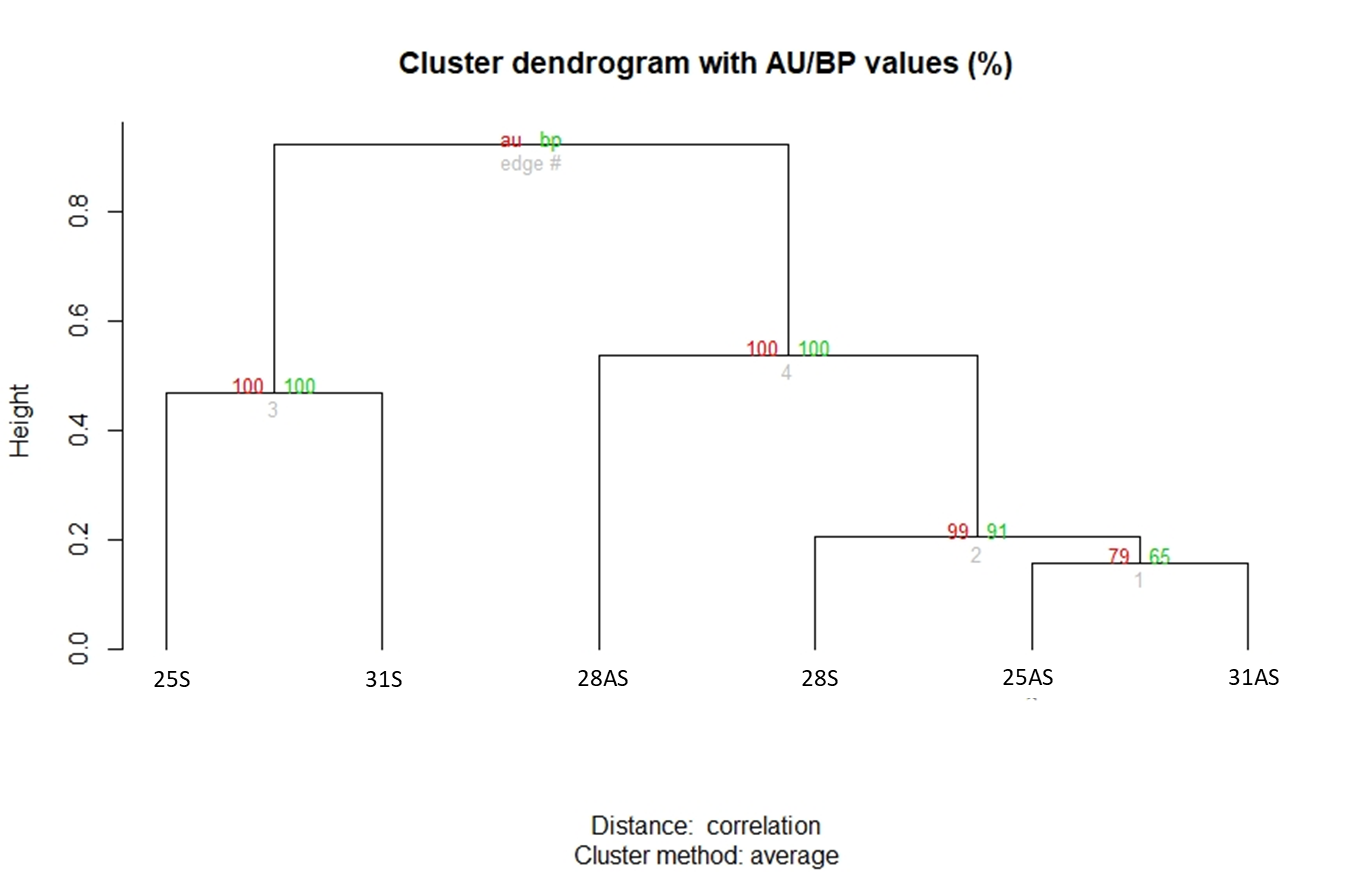


Figure S2 – Venn diagram of S; symbiotic polyps and AS, apo-symbiotic common genes. The diagram includes only the common genes from each treatment (340 common genes of apo-symbiotic and 632 common genes of control groups, see Figure 3). The numbers of up- (red arrows) and down- (green arrows) regulated genes are indicated within each field, as regard to the examined groups 28AS/25S, 31AS/25AS 28S/25S, and 31S/25S. Figure includes data of number of genes in common and unique groups.


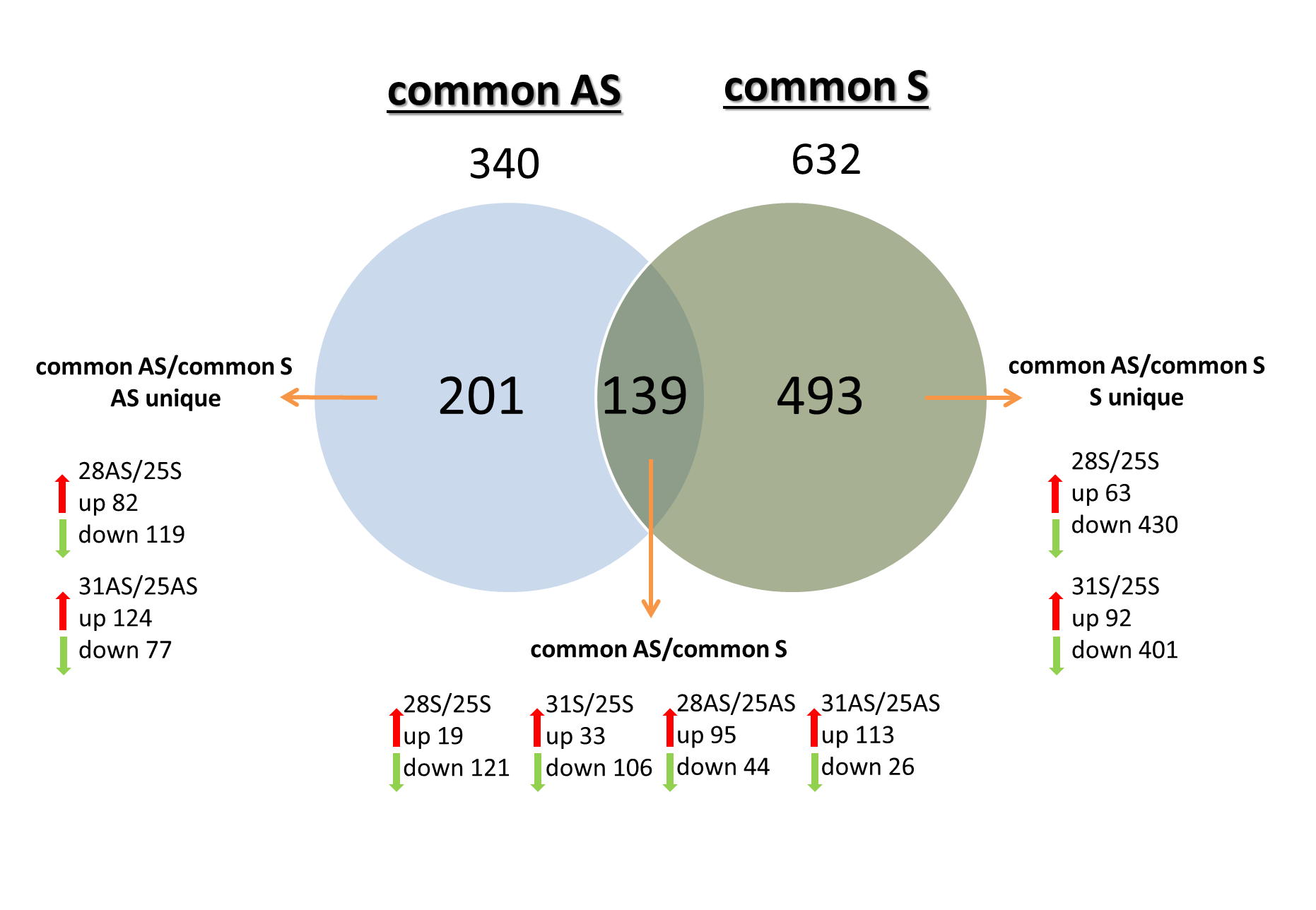


Table S1 – Number of symbiont cells and chlorophyll measurements in representative fragments of each treatment, following one year in light/darkness conditions.

| **treatment** | **Zoox_ (#)** | **Chlorophyll per ml^-3^ (µg_ml^-3^)** | **Chl a_zoox-1** |
| --- | --- | --- | --- |
| light | 221,333,333.3 | 14.23 | 6.96E-07 |
| light | 140,533,333.3 | 27.50 | 1.71E-06 |
| light | 94,933,333.3 | 7.20 | 1.22E-06 |
| light | 77,333,333.3 | 19.53 | 2.51E-06 |
| Darkness | 0 | -0.08 | 0 |
| Darkness | 0 | 0.56 | 0 |
| Darkness | 0 | 0.18 | 0 |
| Darkness | 0 | 0.05 | 0 |

References

Suzuki, R., & Shimodaira, H. (2006). Pvclust: An R package for assessing the uncertainty in hierarchical clustering. *Bioinformatics*, *22*(12), 1540–1542. doi: 10.1093/bioinformatics/btl117
